# Paper spray mass spectrometry for rapid toxicology targeted screening of emergency department samples: a research study

**DOI:** 10.1093/jat/bkag060

**Published:** 2026-07-08

**Authors:** Stefania Boccuzzi, David Cowan, Paul I Dargan, Edward Goucher, Vincenzo Abbate

**Affiliations:** Department of Toxicology & Forensic Science, King’s College London, London, SE1 9NH, United Kingdom; Department of Toxicology & Forensic Science, King’s College London, London, SE1 9NH, United Kingdom; Clinical Toxicology, Faculty of Life Sciences and Medicine, King’s College London, London, SE1 9NH, United Kingdom; Clinical Toxicology, Guy’s and St Thomas' NHS Foundation Trust and King’s Health Partners, London, SE1 7EH, United Kingdom; Thermo Fisher Scientific, San Jose, CA 95134, United States; Department of Toxicology & Forensic Science, King’s College London, London, SE1 9NH, United Kingdom

## Abstract

Acute recreational drug toxicity (ARDT) is a frequent cause of emergency department (ED) presentations, yet comprehensive toxicological testing by chromatographic techniques is rarely conducted due to limited availability and resource-intensive workflows. Consequently, initial clinical decisions are made without analytical confirmation. Paper spray-mass spectrometry (PS-MS) may offer an alternative approach for targeted clinical toxicology screening owing to its rapid and simplified analysis of biological samples. Serum donor samples from patients presenting to the ED with suspected ARDT were analyzed using PS-MS and liquid chromatography-tandem mass spectrometry (LC-MS/MS) for method comparison. Targeted screening and quantitative performance were evaluated using pooled diagnostic metrics, orthogonal regression, per-analyte % bias and Bland-Altman analyses. PS-MS was subsequently applied to a larger screening-only cohort to assess analytical performance, detection frequency, and concentration distributions across a clinically relevant drug panel. Across 114 research samples, PS-MS demonstrated high targeted screening performance, with a pooled sensitivity of 82.7%, specificity and positive predictive value of 100%, and negative predictive value of 98.2%. Quantitative comparison showed strong correlation between methods, with proportional bias at higher concentrations and no single analyte influencing the agreement metrics. In the screening-only cohort (*n* = 99), PS-MS detected and quantified a broad range of analytes, including evidence of polydrug use. Several low-frequency analytes were not observed within the analyzed serum donor sample cohort. PS-MS may provide a fast and specific targeted screening approach for ARDT in ED samples, delivering analytically informative toxicological data. While not a replacement for confirmatory testing or a validated in vitro diagnostic assay, this research study into PS-MS represents a valuable complementary tool to support downstream confirmatory testing.

## Introduction

Acute recreational drug toxicity (ARDT) is a frequent cause of hospital presentations, representing a substantial burden on acute healthcare services. In England, 9690 hospital admissions related to drug poisoning were recorded between April 2022 and March 2023 [1]; this likely underestimates the true burden due to limitations in hospital coding systems, and as many cases of ARDT are assessed, treated, and discharged from the emergency department (ED) without inpatient admission [2]. Timely identification of the substances involved can support diagnosis, risk stratification, and clinical management, particularly where clinical features are non-specific or where polydrug exposure is suspected [3, 4]. However, the time-critical nature of emergency care often contrasts with the availability and turnaround time of comprehensive toxicology testing. Consequently, early ED management decisions are frequently made with limited analytical information, relying primarily on clinical assessment of the presenting features and self-reported patient history of the drug(s) involved [5, 6].

Liquid chromatography-tandem mass spectrometry (LC-MS/MS) is highly regarded for toxicological analysis due to its sensitivity, specificity, and ability to quantify a broad range of compounds [7, 8]; however, implementation in clinical scenarios may be limited by instrument availability and resource-intensive sample preparation and data processing [4]. When instrumentation is available, LC-MS/MS workflows are incompatible with the initial decision-making window in the ED, as analytical run times and data interpretation contribute to extensive turnaround times [6].

Alternative screening approaches, such as immunoassays, are widely available and offer rapid results with minimal sample preparation, but their analytical scope is limited by restricted analyte coverage, variable sensitivity, and susceptibility to cross-reactivity [9–11]. These constraints are particularly relevant for recreational drug use, where patterns of exposure are heterogeneous and continuously evolving [12]. As such, immunoassays alone cannot reliably address the analytical needs associated with suspected ARDT, highlighting a broader gap between rapid screening capability and comprehensive toxicological analysis.

**Figure 1:**
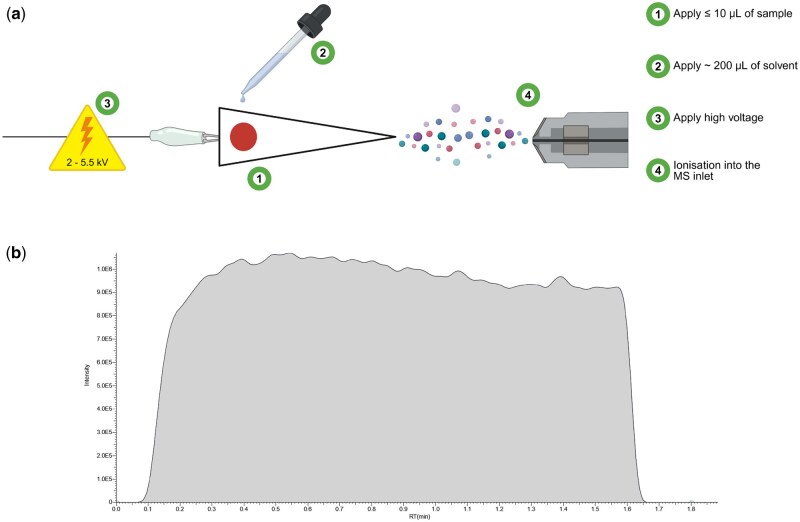
Schematic of the paper spray ionization mechanism for the analysis of a biological matrix spot (a) and the resulting data output in the form of a representative chronogram (b). Figure (a) was generated using BioRender. Reproduced from Boccuzzi *et al*. [15], licensed under Creative Commons Attribution 4.0 International (CC BY 4.0).

Paper spray ionization is an ambient ionization technique that enables direct analysis of biological matrices from a paper substrate with minimal sample preparation. A small volume of sample (≤ 10 μL) is deposited onto a triangular paper substrate and allowed to dry. Upon solvent (≤ 200 μL) and high-voltage (3–5 kV) application, analytes are extracted by capillary action and ionized directly from the paper tip, generating a time-resolved analyte response, termed a chronogram, within 2 minutes per sample [13, 14] (Fig. 1). In contrast to chromatography, paper spray ionization offers analysis times in minutes and requires microlitre-scale sample volumes, reducing consumable use, analytical turnaround time, and overall cost per analysis. Subsequent developments such as coupling to tandem mass spectrometry (PS-MS) and the automated Thermo Scientific™ VeriSpray™ Paper Spray Ionization Source have improved workflow standardization and reproducibility while preserving speed and minimal sample handling of research samples. Accordingly, PS-MS is an attractive candidate for rapid analytical targeted screening workflows aligned with the time constraints of acute toxicology research. Previous studies have demonstrated the feasibility of PS-MS for detecting drugs of abuse encountered in clinical toxicology directly from blood and plasma [16–20]. However, much of the existing literature is limited to analytical validation using spiked samples or proof-of-concept applications. A notable exception, Zimmermann-Federle *et al.* [21], employ integrated solid-phase extraction-based workflows to authentic ED patient samples, however, this relies on sample preparation strategies that are unlikely to be available within ED settings.

The aim of this study was to compare PS-MS with LC-MS/MS for the analysis of research donor samples collected from patients presenting to the ED with suspected ARDT. A targeted panel of 13 analytes was selected based on routine antemortem toxicology testing and input from consultant toxicologists regarding substances of importance in UK acute care. By evaluating analytical agreement, targeted screening performance, and applicability across a clinically informed analyte panel, this research assesses the suitability of PS-MS as an alternative targeted screening tool to support early clinical decision-making.

## Materials and methods

### Materials and chemicals

6-monoacetylmorphine (6-MAM), 6-MAM-d3, alprazolam, alprazolam-d5, benzoylecgonine (BZE), BZE-d3, buprenorphine, buprenorphine-d4, cocaine, cocaine-d3, codeine, codeine-d3, diazepam, diazepam-d5, methylenedioxymethamphetamine (MDMA), MDMA-d5, methadone, methadone-d3, methamphetamine, methamphetamine-d5, zopiclone, zopiclone-d4 (1 mg/mL solution), 2-ethylidene-1,5-dimethyl-3,3-diphenylpyrrolidine (EDDP), EDDP-d3 (1 mg/mL as perchlorate solution), and ketamine-d4 (1 mg/mL as HCl salt solution) were purchased from Sigma-Aldrich (Gillingham, UK). Ketamine (1 mg/mL as HCl salt solution) was donated from TICTAC Communications (London, UK). Stock solutions were diluted in methanol (MeOH) to 25–200 µg/mL and stored at −40°C. LC-MS grade MeOH, acetonitrile (ACN), and formic acid were purchased from Fisher Scientific (Loughborough, UK). Ultrapure water (18.2 MΩ) was delivered by an ELGA PureLab^®^ Chorus 1 system. VeriSpray Paper Spray sample plates were contributed by Thermo Scientific (San Jose, CA, USA). A pre-mixed multi-compound interference panel was donated from the Drug Control Centre (London, UK) containing structurally related, isobaric, and toxicologically relevant compounds; full panel composition is provided in Supplemental Table 1 (see online supplementary material).

### ARDT patient samples

213 serum donor samples were collected from a hospital ED. Of these, 114 were used to evaluate quantitative agreement between PS-MS and LC-MS/MS, and 99 were used to assess PS-MS as a standalone targeted screening approach reflective of its intended clinical application. Inclusion criteria comprised living adult patients presenting to the ED with suspected ARDT, from whom surplus serum obtained during routine clinical blood sampling was available. Samples were collected opportunistically between February 2023 and July 2023. Upon receipt, 45 µL of each serum sample was aliquoted into Starlab 0.5 mL TubeOne^®^ Microcentrifuge Tubes and stored at −80°C. For analysis, samples were spiked with 5 µL of internal standard (ISTD) (Supplemental Tables 2 and 3—see online supplementary material) and vortexed.

### Donor plasma preparation

Drug-free whole blood was collected from 10 healthy donors into K_2_ EDTA Vacutainer^®^ tubes and centrifuged at 2500 rpm using an Eppendorf 5702 centrifuge for 20 minutes. 40 µL of plasma was aliquoted into microcentrifuge tubes and stored at −80°C. For method validation, calibration, and quality control (QC) samples, samples were spiked with 10 µL of analyte/ISTD mix (Supplemental Tables 2 and 3—see online supplementary material), and vortexed.

### Paper spray-mass spectrometry (PS-MS) analysis

Donor plasma (5 µL, spiked with analytes and ISTD) and serum (5 µL, spiked with ISTD only) samples were pipetted onto VeriSpray sample plates containing 24 triangular Whatman ET31 paper strips and dried under ambient laboratory conditions for 15 minutes. Sample analysis was performed using a VeriSpray Paper Spray Ion Source coupled to a Thermo Scientific™ TSQ Altis™ Plus Triple Quadrupole mass spectrometer. The spray solvent (ACN/H_2_O/formic acid, 90:10:0.1 v/v/v) was dispensed as follows: 10 µL with a 3 s delay, 10 µL/s for 4 seconds, 10 µL every 3 seconds for 9 seconds, 10 µL every 5 seconds for 15 seconds. A 4.5 kV spray voltage was applied. Data were acquired in positive ionization mode using selected reaction monitoring (SRM) with four transitions per analyte (Supplemental Table 4—see online supplementary material) for a run time of 2 minutes. Key variables included Q1 and Q3 resolution of 0.7 Da FWHM, ion transfer tube temperature 325 °C, and CID gas pressure 1.5 mTorr using argon.

### LC-MS/MS analysis

For protein precipitation, 150 µL of MeOH/ACN/formic acid (75:25:0.1 v/v/v) was added to each sample, vortexed for 3 minutes, refrigerated for 30 minutes, then centrifuged at 10 000 × g for 10 minutes. The supernatant was transferred to 96-well collection plates and evaporated to dryness under nitrogen for 30 minutes without applied heating. Samples were reconstituted in 50 µL of mobile phase A and vortexed for 1 minute prior to LC-MS/MS injection.

Samples were analyzed using the Thermo Scientific™ Tox Explorer™ Collection LC-MS/MS method on a Thermo Scientific™ Vanquish™ Flex Binary UHPLC coupled to a TSQ Altis Plus Triple Quadrupole mass spectrometer. 10 µL of sample was injected onto a Thermo Scientific™ Accucore™ Phenyl Hexyl column (100 × 2.1 mm, 2.6 µm) maintained at 40 °C, with a binary mobile phase consisting of 2 mM ammonium formate and 0.1% formic acid in water (A) and 2 mM ammonium formate and 0.1% formic acid in ACN/MeOH/H_2_O (50:50:1, v/v/v) (B). The gradient operated at 0.5 mL/minute: 1% B held for 1 minute, increased to 99% B over 9 minutes, held for 1.5 minutes, and returned to 1% B for 4 minutes. The total run time was 15.5 minutes. Ionization was performed using a Thermo Scientific™ OptaMax™ NG H-ESI source with nitrogen at ∼5.6 L/minute (sheath gas) and ∼8 L/minute (auxiliary gas). MS detection was performed in positive ionization mode using SRM, with two transitions per analyte (Supplemental Table 5—see online supplementary material). Other key conditions were a spray voltage of 3.5 kV, Q1 and Q3 resolution of 0.7 Da FWHM, ion transfer tube temperature of 325 °C, and CID gas pressure of 2 mTorr using argon.

### Data acquisition and processing

Sequence set-up, acquisition, and data analysis were conducted using Thermo Scientific™ XCalibur™ 4.5 and Thermo Scientific™ TraceFinder™ software. Quantification was based on analyte-to-ISTD peak area ratios. PS-MS chronogram areas were integrated using a fixed run time window. Compound identification required the presence of all monitored SRM transitions. A positive result was only reported when all ion ratios were within predefined acceptance criteria, in accordance with World Anti-Doping Agency (WADA) guidelines [22]: tolerance windows of ± 10 percentage points (absolute) for ions > 50%–100%, ± 20% (relative) for > 25%–50%, and ± 5 percentage points (absolute) for 1%–25% relative to the base peak. All Thermo Fisher Scientific instruments, consumables, and software referenced in this study are designated for General Laboratory Use only and are not intended or validated for in vitro diagnostic procedures.

### Method validation

Method validation was performed in accordance with ANSI/ASB 036 guidelines [23]. Five 8-point calibration curves and QC samples were prepared depending on the analytical method (Supplemental Tables 2 and 3—see online supplementary material), with 1/x^2^ weighting selected following evaluation of calibration performance across the analytical range, to assess linearity, limit of detection (LOD) (Equation 1), limit of quantification (LOQ) (Equation 2), bias (%) and precision (% CV). The lower limit of quantification (LLOQ) was identified as the lowest calibrator (calibrator 1) meeting predefined acceptance criteria for bias (± 20%), precision (≤ 20%), and ion ratio tolerances.


(1) $$\mathrm{LOD}\;(\mathrm{ng}/\mathrm{mL})\;=\;\frac{\left(3.3\;\times \;s_{y}\right)}{{\mathrm{Avg}}_{m}}\;(1)$$

**Equation 1**. LOD estimation calculation based on the standard deviation of y-intercept (s_y_) and average slope (Avg_m_) of three calibration runs.


(2) $$\mathrm{LOQ}\;(\mathrm{ng}/\mathrm{mL})\;=\;\frac{\left(10\;\times \;s_{y}\right)}{{\mathrm{Avg}}_{m}}(2)$$

**Equation 2**. LOQ estimation calculation based on the standard deviation of y-intercept (s_y_) and average slope (Avg_m_) of three calibration runs.

Carryover was assessed using blank biological matrix after the highest calibrator from 10 donor spots. Interference was evaluated against 78 analytes (Supplemental Table 1—see online supplementary material), and by analyzing blank, analyte-fortified, and ISTD-fortified biological matrices. Matrix effects (ME %) were assessed according to Matuszewski *et al.* (2003) [24]. Samples were diluted 1:5 with blank biological matrix in triplicate to assess dilution integrity. Processed sample stability was assessed for up to 48 hours under photoprotective conditions at room temperature (PS-MS) and 10 °C in the autosampler (LC-MS/MS).

### Data analysis and statistical methods

All statistical analyses were performed using Microsoft Excel (Version 16.041.1) and Minitab Statistical Software (Version 21.4.3.0). To assess matrix-related differences, supportive analyses of ISTD response were performed. Two-sample t-tests and F-tests were applied to the median log_10_ of ISTD response across analytes to compare responses and variance between plasma and serum within each analytical method. Normality was assessed using Anderson–Darling tests. % CV was calculated to measure signal variability. Blank serum and ISTD spiked neat, plasma, and serum samples were used to evaluate endogenous interference and ionization effects, informed by ANSI/ASB 036 guidelines [23].

For LC-MS/MS and PS-MS comparisons, true positive (TP), true negative (TN), false positive (FP), and false negative (FN) was calculated using LC-MS/MS as the reference method. Sensitivity, specificity, positive predictive value (PPV), and negative predictive value (NPV) were calculated using standard definitions [25, 26]. Analytes were classified as positive when concentrations exceeded the lowest validated calibrator (defined as the LLOQ) and all ion ratios satisfied predefined acceptance criteria. Detections below the LLOQ and/or failing ion ratio acceptance criteria were classified as negative for performance calculations. Quantitative agreement between PS-MS and LC-MS/MS was assessed using orthogonal regression and least squares fit of paired concentration measurements. Bland-Altman plots assessed systematic bias and limits of agreement. Additionally, relative bias (%) for each paired result was calculated to provide a measure of the direction and magnitude of deviation between methods at the individual analyte level, defined in Equation 3.


(3) $$\mathrm{Relative}\;\%\;\mathrm{bias}\;\mathrm{per}\;\mathrm{paired}\;\mathrm{result}\;=\;\frac{\mathrm{PS}-\mathrm{MS}\;\mathrm{concentration}-\mathrm{LC}-\mathrm{MS}/\mathrm{MS}\;\mathrm{concentration}}{\mathrm{LC}-\mathrm{MS}/\mathrm{MS}\;\mathrm{concentration}}\;\times \;100\;(3)$$

**Equation 3**. Relative % bias calculation based per paired result of the 114 serum donor samples analyzed by PS-MS and LC-MS/MS.

Per-analyte median relative bias (%) summaries were calculated for compounds with ≥ 5 paired measurements to ensure robustness of summary statistics. Compounds with fewer observations were not included in formal per-analyte summaries due to limited reliability of central tendency estimates.

For PS-MS targeted screening-only, detection frequency by analyte, distribution of the number of drugs detected per sample, and summary concentration statistics (median, minimum, maximum) were assessed.

## Results

### Method validation

Across both analytical techniques, calibration exhibited strong linearity and precision across all analytes and methods, supported by good fit to the linear model (R^2^ values > 0.97), large F-statistics, and small residual errors. Bias was between −15% and 10%, and within-run and between-run % CV were within 22%. Analyte and method-dependent LODs ranged from 1.3 to 49 ng/mL. No carryover was observed. No evidence of isobaric or structurally related interference was observed for the compounds evaluated, with no significant signal detected in the corresponding SRM transitions of the target analytes. ME % ranged from −78% to −11% without adversely impacting quantification. Dilution integrity bias and precision were similar to those obtained without dilution. Samples were stable for 48 hours (Supplemental Tables 6 and 7—see online supplementary material).

### Assessment of plasma and serum matrix effects

Comparison of ISTD responses indicated no observable difference between plasma and serum for PS-MS (*P* = .09), and an observable difference for LC-MS/MS (*P* = .01). Variance between matrices was similar for both techniques (*P* = .40 for PS-MS; *P* = .36 for LC-MS/MS) and no deviation from normality was observed (*P* > .05). % CV was similar between serum and plasma across PS-MS and LC-MS/MS. Blank serum showed no detectable endogenous interference. Ionization effects were consistent between matrices for both techniques (Supplemental Table 8—see online supplementary material).

### Analytical comparison—PS-MS and LC-MS/MS

PS-MS demonstrated high targeted screening performance when benchmarked against LC-MS/MS as the reference analytical method (Table 1); analyte-specific screening performance metrics for the target panel are summarized in Supplemental Table 9 (see online supplementary material). 66 of 114 samples were positive for at least one analyte, corresponding to 81 positive analyte-sample occurrences, including quantified results and detections below the LLOQ. Among the 66 positive samples, both PS-MS and LC-MS/MS generated detectable signals for all analytes; however, PS-MS did not meet the predefined quantification criterion for a subset of low-level detections.

**Table 1:** Pooled targeted screening performance metrics for PS-MS relative to LC-MS/MS (*n* = 822).

True positives	67
True negatives	741
False positives	0
False negatives	14
Pooled sensitivity	82.7% (95% CI: 73.1% to 89.4%)
Pooled specificity	100% (95% CI: 99.5% to 100%)
Pooled PPV	100% (95% CI: 94.6% to 100%)
Pooled NPV	98.2% (95% CI: 97% to 98.9%)

Targeted screening performance metrics were calculated across 822 analyte-sample decisions, reflecting detected and non-detected analytes. No FPs were observed, and a pooled sensitivity of 82.7% (95% CI: 73.1 to 89.4%) reflects the ability of PS-MS to identify analytes present in patient samples within the predefined reporting criteria. The pooled NPV of 98.2% (95% CI: 97 to 98.9%) reflects a low frequency of 14 FNs observed. Importantly, these FNs did not reflect complete absence of PS-MS signal; however, the responses fell below the LLOQ and did not satisfy the ion ratio acceptance criteria required for positive classification. In these cases, the corresponding LC-MS/MS results met the predefined reporting criteria for positive classification, resulting in FN classifications for PS-MS within the method comparison. The FNs predominantly occurred for diazepam (*n* = 7), followed by BZE (*n* = 5) and cocaine (*n* = 2). Four analytes (6-MAM, buprenorphine, EDDP, and zopiclone) did not produce detectable signal above the LOD by either PS-MS or LC-MS/MS in any analyzed sample.

**Figure 2:**
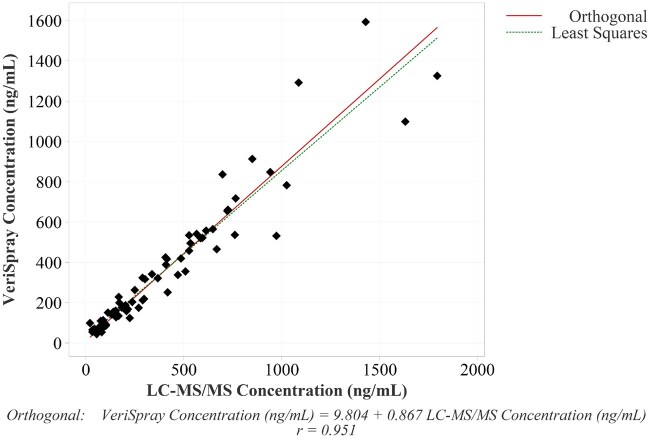
Orthogonal and least-squares regression (error variance = 0.95) comparing PS-MS and LC-MS/MS concentrations in the method-comparison cohort of 114 serum donor samples. Each data point represents a single analyte measurement within a sample; therefore, samples containing multiple detected compounds contribute multiple data points. Data are pooled across all analytes to provide an overall assessment of quantitative agreement between methods. Generated using Minitab Statistical Software, Version 21.4.3.0.

Quantitative agreement between PS-MS and LC-MS/MS concentrations was assessed using orthogonal regression analysis of paired detections, as shown in Fig. 2, which presents pooled agreement across all analytes. A strong linear relationship between the two analytical methods (Pearson *r *= 0.951) indicated that PS-MS responses tracked relative concentration changes measured by LC-MS/MS. The intercept indicates no fixed offset at low concentrations (9.8 ng/mL, 95% CI: −27.4 to 47.0), while the slope of 0.867 (95% CI: 0.800 to 0.935) demonstrates proportional bias, with PS-MS underestimating concentrations relative to LC-MS/MS at higher levels, corresponding to an approximate 13% lower response per unit increase in concentration. This trend was observed across the dataset, reflecting a predictable compression of the concentration range rather than random disagreement between methods.

**Figure 3:**
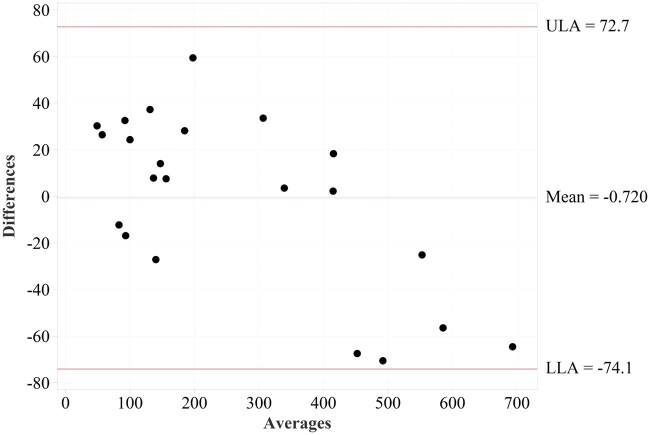
A representative Bland-Altman plot for methamphetamine comparing PS-MS and LC-MS/MS concentrations. ULA, upper limit of agreement; LLA, lower limit of agreement. Generated using Minitab Statistical Software, Version 21.4.3.0.

Bland-Altman analysis demonstrated close agreement between PS-MS and LC-MS/MS at lower concentrations, with increasing negative bias and dispersion at higher concentrations, and most paired measurements remaining within the limits of agreement, as shown by the representative plot for methamphetamine in Fig. 3. Similar trends were observed across other commonly detected compounds. These findings indicate a concentration-dependent difference between methods, in which PS-MS increasingly underestimates concentration relative to LC-MS/MS, consistent with the proportional bias observed in orthogonal regression analysis.

Per-analyte relative bias (%) analysis (Table 2) demonstrated broadly consistent trends across the most frequently detected compounds, with predominantly negative bias observed. Some variability between analytes was evident, however, no single analyte disproportionately influenced the pooled agreement observed in Fig. 2.

**Table 2:** Per-analyte median relative bias (%) between PS-MS and LC-MS/MS for compounds with ≥ 5 paired measurements.

Analyte	Paired samples (*n*)	**Median relative bias (%)** ^a^
BZE	17	−8.0
Diazepam	5	−23.7
Ketamine	11	−23.9
MDMA	6	−10.8
Methamphetamine	22	4.7

a Median values are reported due to non-normal distribution and small sample sizes.

### PS-MS targeted screening of patient samples

68 of 99 samples were positive for at least one analyte, corresponding to 91 detections. The distribution of detected (< LLOQ, > LOD) and quantified (≥ LLOQ) findings is summarized in Table 3. Of the 91 detections, 21 (23.1%) were detected below the LLOQ, predominantly for diazepam, followed by methadone, zopiclone, and EDDP. The remaining 70 detections (76.9%) were quantifiable, with methamphetamine and BZE most frequently quantified, followed by diazepam and MDMA. Four analytes included in the panel (6-MAM, alprazolam, buprenorphine, and cocaine) were not detected in any sample within this cohort.

**Table 3:** Distribution of PS-MS detections and quantifications by analyte in the targeted screening-only cohort.

Analyte	**Detected** ^a^		**Quantified** ^b^	
	Number of samples	% of samples	Number of samples	% of samples
BZE	0	0.0%	20	20.2%
Codeine	0	0.0%	1	1.0%
Diazepam	13	13.1%	10	10.1%
EDDP	1	1.0%	2	2.0%
Ketamine	0	0.0%	5	5.1%
MDMA	0	0.0%	8	8.1%
Methadone	5	5.1%	4	4.0%
Methamphetamine	0	0.0%	20	20.2%
Zopiclone	2	2.0%	0	0.0%

a Detected by PS-MS at concentrations above the limit of detection but below the lower limit of quantification (> LOD, < LLOQ).

b Quantified by PS-MS at concentrations at or above the lower limit of quantification (≥ LLOQ).

**Figure 4:**
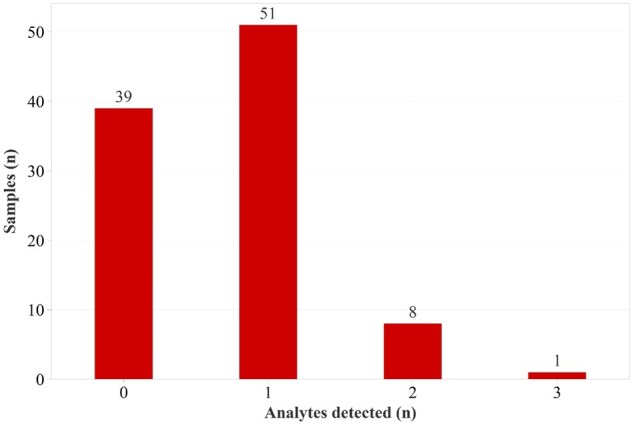
Distribution of the number of analytes detected per sample in the PS-MS targeted screening cohort of 99 serum donor samples. Generated using Minitab Statistical Software, Version 21.4.3.0.

Quantified analytes spanned median concentrations ranging from 65.3 to 726 ng/mL and minimum and maximum observed values extending from 30 to 2140 ng/mL (Supplemental Table 10—see online supplementary material). Most positive samples contained a single detected analyte; polydrug use was detected in a 9.1% of positive samples (Fig. 4).

## Discussion

This research study assessed the suitability of PS-MS as a rapid targeted screening approach for drug detection in donor serum samples from patients presenting to the ED with ARDT, using LC-MS/MS as the reference method, consistent with its role as the gold standard for confirmatory toxicological testing. Quantitative comparison therefore reflects agreement relative to an established reference method, rather than absolute accuracy against a known true value. The standardized Tox Explorer Collection LC-MS/MS workflow provided a consistent comparator method for evaluation of PS-MS targeted screening performance across a broad drug panel. While LC-MS/MS remains essential for definitive quantification, the shorter run time of PS-MS enables analytical signal acquisition within minutes of sample preparation. This temporal advantage is central to the possible utility of PS-MS as a targeted screening tool in acute care.

In routine ED practice, availability of surplus biological matrix is determined by clinical workflows rather than study design. Consequently, analytical approaches capable of accommodating variability in available sample matrices are advantageous in real-world clinical implementation. Although calibration and QC materials were prepared in plasma while donor patient samples were serum, supportive matrix comparison studies demonstrated comparable ISTD behavior, signal variability, and ion suppression profiles between matrices under the applied conditions, suggesting limited matrix-dependent contribution to the observed inter-method differences. Importantly, these findings did not translate into FP results and had minimal impact on targeted screening performance metrics.

The targeted screening performance observed in this study reflects the hierarchy between PS-MS and LC-MS/MS observed in literature, with LC-MS/MS supporting higher sensitivity and robust quantification and PS-MS showing good qualitative agreement and selectivity [16, 27, 28]. The absence of FP results is a critical requirement for ED targeted screening where incorrect attribution of drug exposure may lead to inappropriate clinical decisions. The applied reporting criteria aligned with validated analytical thresholds. Consequently, FN classifications occurred where analyte-related PS-MS signal was observed but did not satisfy the predefined reporting criteria for positive classification relative to LC-MS/MS. These findings suggest that the reported sensitivity reflects analytical detectability as well as the application of conservative reporting criteria designed to support analytical confidence. As such, the observed FN rate should be interpreted in the context of the reporting thresholds applied rather than as complete failure of analyte detection by PS-MS.

Two FNs for BZE represented complete non-detection in PS-MS, while LC-MS/MS quantified these samples at low concentrations of 55.3 ng/mL and 68.6 ng/mL. Such values lie at the lower end of ranges reported in toxicology [29–32] and are subject to interpretive uncertainty in the absence of sampling time and dose information. While these concentrations may reflect early or late sampling relative to cocaine use, very early sampling has been shown to yield detectable parent cocaine [33], which was not observed in these cases. Accordingly, the analytical sensitivity limitations observed for PS-MS at very low concentrations are unlikely to translate into clinically significant diagnostic gaps when interpreted within routine ED targeted screening workflows.

The proportional bias observed is consistent with the fundamental differences between PS-MS and LC-MS/MS. PS-MS performs direct ionization from a dried matrix without chromatographic separation, making it more susceptible to concentration-dependent ionization behavior and matrix-driven variability [34, 35] whereas LC-MS/MS separation facilitates temporal isolation of analytes and enables more effective mitigation of matrix effects prior to isolation [36, 37].

The lack of a fixed bias at low concentrations supports the reliability of PS-MS for relative concentration assessment within the clinically relevant low-to-moderate range. Increased dispersion and negative bias at higher concentrations therefore reflect predictable method behavior rather than analytical instability, reinforcing that PS-MS should not provide interchangeable quantitative results with LC-MS/MS.

Beyond quantitative agreement, patterns of parent drug and metabolite detection provide additional insight into the interpretation of toxicological findings. For example, detection of methadone in the absence of its primary metabolite EDDP may reflect low concentrations approaching the LOQ, recent administration prior to substantial metabolism [29], or interindividual variability in metabolic rate [38]. Conversely, the presence of BZE in the absence of cocaine is consistent with the known rapid hydrolysis of cocaine to its inactive metabolites [29]. The inclusion of parent compounds and metabolites, whether inactive or not, in the analytical panel was intentional to enable insight into exposure patterns that would not be apparent from parent drug detection alone, such as timing of exposure and metabolic progression to inform potential clinical relevance. Furthermore, the observed prevalence of polydrug exposure and commonly detected substances was broadly consistent with previous ARDT literature [6], although regional and temporal drug trends remain dynamic.

Evaluation of PS-MS in a targeted screening-only cohort for the detection of a broad range of drugs and identification of polydrug exposure relevant to ARDT reflect real-world complexity typical of ED presentations. Importantly, PS-MS could provide laboratory scientists and clinical researchers with structured toxicological insight in settings where comprehensive LC-MS/MS analysis is unavailable or delayed. In the acute setting, such information may support clinical decision-making by aiding risk stratification, informing the need for admission versus discharge, and guiding the level and duration of monitoring, particularly in cases of uncertain history or suspected polydrug exposure [3, 4]. When quantification is not achieved, qualitative or semi-quantitative detection offers a meaningful evidentiary basis to contextualize patient presentation, particularly compared with absence of analytical data. Notably, clinical management in acute toxicity is rarely dependent on the availability of a specific antidote but instead guided by assessment of severity and risk of deterioration, for which timely toxicological information remains valuable. Unlike immunoassay screening approaches, PS-MS does not introduce class-based identification or cross-reactivity effects. While class-level detection may support rapid initial treatment decisions in select scenarios, it lacks specificity and does not provide quantitative information, limiting its utility for risk stratification and ongoing clinical management. In contrast, PS-MS enables compound-specific identification supported through targeted method development, the use of matched ISTDs, and stringent ion ratio acceptance criteria, such as those outlined by WADA [22], to support robustness in the absence of chromatographic separation.

A strength of this research study is the use of authentic patient donor samples analyzed under deliberately constrained analytical conditions reflective of routine ED practice, where true concentrations are unknown and controlled spiking experiments are not representative of clinical workflows. Analysis was performed without offline or embedded sample extraction, relying on minimal sample preparation and direct analysis from biological matrices. Additionally, a targeted acquisition prioritizes sensitivity for predefined, toxicologically relevant analytes over broad non-targeted detection to reflect clinically relevant compounds spanning multiple drug classes and physicochemical properties. The simplified PS-MS platform is adaptable, allowing for straightforward expansion to include emerging substances without fundamental changes to the analytical workflow. The combined evaluation of method comparison and targeted screening-only cohorts allows separation of analytical performance from routine utility, strengthening interpretation of PS-MS behavior in clinical contexts. Maintaining the use of low sample volumes (≤ 45 μL) across both methods ensured that analytical performance was not dependent on access to large sample volumes, reflecting realistic clinical conditions where access to surplus samples may be limited. Although urine toxicology may provide a broader detection window for certain analytes, including compounds preferentially detected following renal excretion, plasma and serum better reflect acute circulating drug exposure at the time of clinical presentation. Furthermore, blood is routinely collected during ED assessment and may be more practical than urine collection in severely intoxicated or clinically unstable patients.

Limitations include reliance on single analytical replicates and analyte-specific case distributions that limited robust sensitivity estimates for less frequently encountered drugs. Despite reflecting routine ED practice, variability in sampling time relative to drug ingestion may have contributed to low-concentration findings near analytical detection thresholds. Additionally, findings may not generalize to compounds with substantially different physicochemical properties or ionization behavior. In the absence of chromatographic separation, a recognized limitation of direct PS-MS methodologies is the potential for interference from isobaric or structurally related compounds. In the present study, specificity was supported through the use of multiple SRM transitions per analyte, matched ISTDs, and stringent ion ratio acceptance criteria. Interference testing included structurally related and potentially isobaric compounds selected to assess the risk of false-positive signal generation within the monitored transitions of the target analyte panel. For example, phenpromethamine, a constitutional isomer of methamphetamine (C_10_H_15_N), was included as a relevant specificity challenge; however, no false-positive methamphetamine signal was observed under the conditions evaluated. Similarly, methamphetamine and phentermine have previously been reported to exhibit analytical interference in certain PS-MS methodologies [39], yet no such interference was observed in the present study. Finally, as sample inclusion depended on availability of surplus biological matrices, some degree of selection bias may have been introduced. While not exhaustive, these findings support the specificity of the PS-MS approach for the compounds evaluated and highlight the effectiveness of optimized SRM transitions and ion ratio criteria in mitigating interference from structurally related species.

## Conclusion

Overall, these research findings support PS-MS as a complementary tool to LC-MS/MS capable of rapid, clinically relevant toxicological targeted screening directly from plasma or serum in an ED setting. By enabling near-immediate assessment of samples, PS-MS has shown robustness against matrix variation and the potential to enhance clinical situational awareness and inform risk stratification during the critical initial phase of patient management. Nevertheless, several analytes within the target panel were not detected in the analyzed cohort, limiting assessment of their real-world screening performance. Future work should explore integration of PS-MS within ED workflows, evaluate performance across broader drug panels, and assess clinical impact on decision-making and patient outcomes. Exploring integration of high-resolution mass spectrometry could build on the present targeted screening approach, enabling broader, non-targeted detection to complement triple quadrupole targeted screening where identification of unexpected or emerging substances is required.

## Supplementary Material

bkag060_Supplementary_Data

## Data Availability

The data underlying this article are available in the article and in its online supplementary material.
