# Supplementary material for: Paper spray mass spectrometry for rapid toxicology targeted screening of emergency department samples: a research study
Source: J Anal Toxicol. 2026 Jul 8;50(7):bkag060. doi: 10.1093/jat/bkag060 (PMC13392471; doi:10.1093/jat/bkag060)
Supplement: bkag060_Supplementary_Data [file bkag060_supplementary_data.zip › jat-26-4849-File007.docx]

| **Supplemental Table 1. Compounds evaluated for analytical interference during QC testing.** | |
| --- | --- |
| Analyte | Concentration (ng/mL) |
| 3-methyl-fentanyl | 10 |
| Adrafinil | 500 |
| AICAR | 100 |
| Altizide | 1,000 |
| Amfepramone | 500 |
| Amphetaminil | 500 |
| Andarine | 10 |
| Bendroflumethiazide | 1,000 |
| Benfluorex | 500 |
| Benzphetamine | 500 |
| Benzthiazide | 1,000 |
| Benzylpiperazine | 500 |
| Butizide | 1,000 |
| Carphedon | 500 |
| Cathine | 500 |
| Clobenzorex | 500 |
| Clomiphene | 200 |
| Cropropamide | 500 |
| Crotethamide | 500 |
| Cyclopenthiazide | 1,000 |
| Cyclothiazide | 1,000 |
| Dexamethasone | 600 |
| Diacetolol | 500 |
| Dimethylamphetamine | 500 |
| Dobutamine | 500 |
| Efaproxiral | 100 |
| Ephedrine | 500 |
| Epitizide | 1,000 |
| Eplerenone | 1,000 |
| Etamivan | 500 |
| Ethylphenylbutylamine | 500 |
| Etilamphetamine | 500 |
| Etilefrine | 500 |
| Exemestane metabolite | 200 |
| Famprofazone | 500 |
| Fenbutrazate | 500 |
| Fencamfamin | 500 |
| Fencamine | 500 |
| Fenetylline | 500 |
| Fenfluramine | 500 |
| Fenproporex | 500 |
| Flunisolide | 300 |
| Fulvestrant | 200 |
| Furfenorex | 500 |
| Heptaminol | 500 |
| Hydroxybroman | 500 |
| Isometheptene | 500 |
| Letrozole metab. | 200 |
| Mefenorex | 500 |
| Mephentermine | 500 |
| Methoxyphenamine | 500 |
| Methylephedrine | 500 |
| Methylhexaneamine | 500 |
| Methylphenidate | 500 |
| Metolazone | 1,000 |
| Nadolol | 500 |
| Nikethamide | 500 |
| Norfenfluramine | 500 |
| Ostarine | 10 |
| Oxilofrine | 500 |
| Pemoline | 500 |
| Pentetrazol | 500 |
| Phenmetrazine | 500 |
| Phenpromethamine | 500 |
| Phentermine | 500 |
| Pholedrine | 500 |
| Piretanide | 1,000 |
| p-OH amphetamine. | 500 |
| Polythiazide | 1,000 |
| Prenylamine | 500 |
| Probenecid | 1,000 |
| Prolintane | 500 |
| Propylhexedrine | 500 |
| Salmeterol | 100 |
| Sibutramine | 500 |
| Strychnine | 500 |
| Trichlormethiazide | 1,000 |
| Tuaminoheptane | 500 |

| **Supplemental Table 2. Summary of analyte-specific concentration ranges, quality control (QC) levels and their corresponding internal standard (ISTD), and ISTD concentrations used for LC–MS/MS.** | | | | | | |
| --- | --- | --- | --- | --- | --- | --- |
| Analyte | Calibration range (ng/mL) | QC concentrations (ng/mL) | | | ISTD | ISTD concentration (ng/mL) |
|  |  | Low | Med | High |  |  |
| 6-MAM | 5 - 200 | 15 | 75 | 150 | 6-MAM-d3 | 100 |
| Alprazolam | 50 - 1,500 | 150 | 750 | 1,200 | Alprazolam-d5 | 750 |
| Buprenorphine | 5 - 200 | 15 | 75 | 150 | Buprenorphine-d4 | 100 |
| BZE | 50 - 1,500 | 150 | 750 | 1,200 | BZE-d3 | 750 |
| Cocaine | 25 - 500 | 75 | 250 | 400 | Cocaine-d3 | 250 |
| Codeine | 25 - 500 | 75 | 250 | 400 | Codeine-d3 | 250 |
| Diazepam | 50 - 1,500 | 150 | 750 | 1,200 | Diazepam-d5 | 750 |
| EDDP | 25 - 500 | 75 | 250 | 400 | EDDP-d3 | 250 |
| Ketamine | 50 - 1,500 | 150 | 750 | 1,200 | Ketamine-d4 | 750 |
| MDMA | 25 - 500 | 75 | 250 | 400 | MDMA-d5 | 250 |
| Methadone | 50 - 1,500 | 150 | 750 | 1,200 | Methadone-d3 | 750 |
| Methamphetamine | 25 - 500 | 75 | 250 | 400 | Methamphetamine-d5 | 250 |
| Zopiclone | 50 - 1,500 | 150 | 750 | 1,200 | Zopiclone-d4 | 750 |

| **Supplemental Table 3. Summary of analyte-specific concentration ranges, quality control (QC) levels and their corresponding internal standard (ISTD), and ISTD concentrations used for PS-MS.** | | | | | | |
| --- | --- | --- | --- | --- | --- | --- |
| Analyte | Calibration range (ng/mL) | QC concentrations (ng/mL) | | | ISTD | ISTD concentration (ng/mL) |
|  |  | Low | Med | High |  |  |
| 6-MAM | 20 - 200 | 45 | 100 | 150 | 6-MAM-d3 | 100 |
| Alprazolam | 50 - 1,500 | 150 | 750 | 1,200 | Alprazolam-d5 | 750 |
| Buprenorphine | 20 - 200 | 45 | 100 | 150 | Buprenorphine-d4 | 100 |
| BZE | 150 - 3,000 | 450 | 1,500 | 2,500 | BZE-d3 | 1,500 |
| Cocaine | 150 - 3,000 | 450 | 1,500 | 2,500 | Cocaine-d3 | 1,500 |
| Codeine | 50 - 1,500 | 150 | 750 | 1,200 | Codeine-d3 | 750 |
| Diazepam | 150 - 3,000 | 450 | 1,500 | 2,500 | Diazepam-d5 | 1,500 |
| EDDP | 25 - 500 | 75 | 250 | 400 | EDDP-d3 | 250 |
| Ketamine | 150 - 3,000 | 450 | 1,500 | 2,500 | Ketamine-d4 | 1,500 |
| MDMA | 25 - 500 | 75 | 250 | 400 | MDMA-d5 | 250 |
| Methadone | 50 - 1,500 | 150 | 750 | 1,200 | Methadone-d3 | 750 |
| Methamphetamine | 25 - 500 | 75 | 250 | 400 | Methamphetamine-d5 | 250 |
| Zopiclone | 50 - 1,500 | 150 | 750 | 1,200 | Zopiclone-d4 | 750 |

| **Supplemental Table 4. Compound specific MS conditions for SRM acquisitions for PS-MS. Quantifier ion transitions are denoted by *.** | | | | | |
| --- | --- | --- | --- | --- | --- |
| Compound | Precursor (m/z) | Product (m/z) | Ion ratios (%) | Collision Energy (eV) | RF Lens (V) |
| 6-MAM | 328.0 | * 165.0 | 100 | 42 | 80 |
| 6-MAM | 328.0 | 191.0 | 26 | 35 | 80 |
| 6-MAM | 328.0 | 193.0 | 41 | 29 | 80 |
| 6-MAM | 328.0 | 211.0 | 57 | 28 | 80 |
| 6-MAM-d3 | 331.0 | * 165.0 | 100 | 42 | 87 |
| 6-MAM-d3 | 331.0 | 181.0 | 23 | 45 | 87 |
| 6-MAM-d3 | 331.0 | 193.0 | 35 | 30 | 87 |
| 6-MAM-d3 | 331.0 | 211.0 | 58 | 28 | 87 |
| Alprazolam | 309.0 | * 165.0 | 100 | 32 | 85 |
| Alprazolam | 309.0 | 205.0 | 76 | 45 | 85 |
| Alprazolam | 309.0 | 274.0 | 42 | 26 | 85 |
| Alprazolam | 309.0 | 281.0 | 14 | 27 | 85 |
| Alprazolam-d5 | 314.0 | * 165.0 | 100 | 31 | 85 |
| Alprazolam-d5 | 314.0 | 210.0 | 78 | 44 | 85 |
| Alprazolam-d5 | 314.0 | 279.0 | 45 | 27 | 85 |
| Alprazolam-d5 | 314.0 | 286.0 | 13 | 28 | 85 |
| Buprenorphine | 468.0 | * 243.0 | 100 | 41 | 118 |
| Buprenorphine | 468.0 | 267.0 | 24 | 50 | 118 |
| Buprenorphine | 468.0 | 396.0 | 74 | 41 | 118 |
| Buprenorphine | 468.0 | 414.0 | 32 | 36 | 118 |
| Buprenorphine-d4 | 472.0 | * 267.0 | 100 | 52 | 99 |
| Buprenorphine-d4 | 472.0 | 400.0 | 39 | 45 | 99 |
| Buprenorphine-d4 | 472.0 | 414.0 | 51 | 37 | 99 |
| Buprenorphine-d4 | 472.0 | 415.0 | 28 | 37 | 99 |
| BZE | 290.0 | * 105.0 | 100 | 31 | 61 |
| BZE | 290.0 | 119.0 | 12 | 29 | 61 |
| BZE | 290.0 | 122.0 | 4 | 30 | 61 |
| BZE | 290.0 | 168.0 | 26 | 20 | 61 |
| BZE-d3 | 293.0 | * 105.0 | 100 | 30 | 62 |
| BZE-d3 | 293.0 | 153.0 | 6 | 24 | 62 |
| BZE-d3 | 293.0 | 171.0 | 4 | 19 | 62 |
| BZE-d3 | 293.0 | 275.0 | 26 | 17 | 62 |
| Cocaine | 304.0 | * 82.0 | 100 | 31 | 64 |
| Cocaine | 304.0 | 105.0 | 12 | 32 | 64 |
| Cocaine | 304.0 | 150.0 | 8 | 26 | 64 |
| Cocaine | 304.0 | 182.0 | 24 | 20 | 64 |
| Cocaine-d3 | 307.0 | * 85.0 | 100 | 32 | 65 |
| Cocaine-d3 | 307.0 | 105.0 | 12 | 33 | 65 |
| Cocaine-d3 | 307.0 | 119.0 | 7 | 31 | 65 |
| Cocaine-d3 | 307.0 | 185.0 | 25 | 20 | 65 |
| Codeine | 300.0 | * 152.0 | 100 | 72 | 77 |
| Codeine | 300.0 | 165.0 | 62 | 46 | 77 |
| Codeine | 300.0 | 199.0 | 83 | 31 | 77 |
| Codeine | 300.0 | 215.0 | 102 | 27 | 77 |
| Codeine-d3 | 303.0 | * 165.0 | 100 | 45 | 80 |
| Codeine-d3 | 303.0 | 183.0 | 59 | 30 | 80 |
| Codeine-d3 | 303.0 | 199.0 | 71 | 30 | 80 |
| Codeine-d3 | 303.0 | 215.0 | 113 | 26 | 80 |
| Diazepam | 285.0 | * 154.0 | 100 | 28 | 91 |
| Diazepam | 285.0 | 193.0 | 63 | 33 | 91 |
| Diazepam | 285.0 | 222.0 | 41 | 28 | 91 |
| Diazepam | 285.0 | 257.0 | 94 | 23 | 91 |
| Diazepam-d5 | 290.0 | * 154.0 | 100 | 29 | 85 |
| Diazepam-d5 | 290.0 | 198.0 | 70 | 33 | 85 |
| Diazepam-d5 | 290.0 | 227.0 | 45 | 28 | 85 |
| Diazepam-d5 | 290.0 | 262.0 | 87 | 23 | 85 |
| EDDP | 278.0 | * 186.0 | 100 | 35 | 82 |
| EDDP | 278.0 | 219.0 | 24 | 43 | 82 |
| EDDP | 278.0 | 234.0 | 37 | 31 | 82 |
| EDDP | 278.0 | 249.0 | 22 | 25 | 82 |
| EDDP-d3 | 281.0 | * 189.0 | 100 | 38 | 78 |
| EDDP-d3 | 281.0 | 219.0 | 24 | 45 | 78 |
| EDDP-d3 | 281.0 | 234.0 | 38 | 32 | 78 |
| EDDP-d3 | 281.0 | 249.0 | 21 | 25 | 78 |
| Ketamine | 238.0 | * 125.0 | 100 | 27 | 40 |
| Ketamine | 238.0 | 179.0 | 54 | 18 | 40 |
| Ketamine | 238.0 | 207.1 | 37 | 15 | 40 |
| Ketamine | 238.0 | 220.1 | 33 | 15 | 40 |
| Ketamine-d4 | 242.0 | * 129.0 | 100 | 28 | 50 |
| Ketamine-d4 | 242.0 | 183.1 | 53 | 18 | 50 |
| Ketamine-d4 | 242.0 | 211.1 | 39 | 15 | 50 |
| Ketamine-d4 | 242.0 | 224.1 | 34 | 16 | 50 |
| MDMA | 194.0 | * 105.0 | 100 | 24 | 37 |
| MDMA | 194.0 | 133.0 | 29 | 20 | 37 |
| MDMA | 194.0 | 135.0 | 31 | 21 | 37 |
| MDMA | 194.0 | 163.0 | 35 | 13 | 37 |
| MDMA-d5 | 199.0 | * 107.0 | 100 | 25 | 39 |
| MDMA-d5 | 199.0 | 135.0 | 37 | 21 | 39 |
| MDMA-d5 | 199.0 | 165.0 | 0 | 14 | 39 |
| MDMA-d5 | 199.0 | 167.0 | 34 | 6 | 39 |
| Methadone | 310.0 | * 105.0 | 100 | 29 | 50 |
| Methadone | 310.0 | 219.0 | 8 | 25 | 50 |
| Methadone | 310.0 | 223.0 | 8 | 22 | 50 |
| Methadone | 310.0 | 265.0 | 34 | 15 | 50 |
| Methadone-d3 | 313.0 | * 105.0 | 100 | 29 | 55 |
| Methadone-d3 | 313.0 | 220.0 | 7 | 25 | 55 |
| Methadone-d3 | 313.0 | 226.0 | 9 | 22 | 55 |
| Methadone-d3 | 313.0 | 268.0 | 36 | 16 | 55 |
| Methamphetamine | 150.0 | * 51.0 | 100 | 64 | 31 |
| Methamphetamine | 150.0 | 65.0 | 20 | 42 | 31 |
| Methamphetamine | 150.0 | 91.0 | 42 | 21 | 31 |
| Methamphetamine | 150.0 | 119.0 | 4 | 12 | 31 |
| Methamphetamine-d5 | 155.0 | * 66.0 | 100 | 41 | 34 |
| Methamphetamine-d5 | 155.0 | 92.0 | 71 | 20 | 34 |
| Methamphetamine-d5 | 155.0 | 121.0 | 0 | 12 | 34 |
| Methamphetamine-d5 | 155.0 | 123.0 | 15 | 6 | 34 |
| Zopiclone | 389.0 | * 112.0 | 100 | 55 | 50 |
| Zopiclone | 389.0 | 217.1 | 53 | 32 | 50 |
| Zopiclone | 389.0 | 245.0 | 38 | 17 | 50 |
| Zopiclone | 389.0 | 345.1 | 36 | 10 | 50 |
| Zopiclone-d4 | 393.2 | * 112.0 | 100 | 55 | 40 |
| Zopiclone-d4 | 393.2 | 217.0 | 56 | 32 | 40 |
| Zopiclone-d4 | 393.2 | 245.0 | 42 | 20 | 40 |
| Zopiclone-d4 | 393.2 | 349.1 | 39 | 10 | 40 |

| **Supplemental Table 5. Compound specific MS conditions for SRM acquisitions for LC-MS/MS. Quantifier ion transitions are denoted by *.** | | | | | | |
| --- | --- | --- | --- | --- | --- | --- |
| Compound | Retention time (min) | Precursor (m/z) | Product (m/z) | Ion ratios (%) | Collision Energy (eV) | RF Lens (V) |
| 6-MAM | 3.59 | 328.2 | * 165.1 | 100 | 38 | 78 |
| 6-MAM | 3.59 | 328.2 | 211.1 | 61 | 26 | 78 |
| 6-MAM-d3 | 3.59 | 331.2 | * 165.1 | 100 | 38 | 77 |
| 6-MAM-d3 | 3.59 | 331.2 | 211.1 | 61 | 26 | 77 |
| Alprazolam | 6.61 | 309.1 | * 205.0 | 100 | 41 | 72 |
| Alprazolam | 6.61 | 309.1 | 281.0 | 87 | 26 | 72 |
| Alprazolam-d5 | 6.59 | 314.1 | * 210.1 | 100 | 44 | 85 |
| Alprazolam-d5 | 6.59 | 314.1 | 286.1 | 85 | 28 | 85 |
| Buprenorphine | 5.81 | 468.3 | * 396.3 | 100 | 40 | 63 |
| Buprenorphine | 5.81 | 468.3 | 414.3 | 91 | 35 | 63 |
| Buprenorphine-d4 | 5.80 | 472.4 | * 400.2 | 100 | 40 | 100 |
| Buprenorphine-d4 | 5.80 | 472.4 | 415.2 | 54 | 35 | 100 |
| BZE | 4.10 | 290.1 | * 105.1 | 100 | 30 | 62 |
| BZE | 4.10 | 290.1 | 167.8 | 39 | 19 | 62 |
| BZE-d3 | 4.10 | 293.1 | * 119.1 | 100 | 30 | 60 |
| BZE-d3 | 4.10 | 293.1 | 171.1 | 8 | 20 | 60 |
| Cocaine | 4.63 | 304.2 | * 82.1 | 100 | 27 | 71 |
| Cocaine | 4.63 | 304.2 | 182.1 | 28 | 20 | 71 |
| Cocaine-d3 | 4.62 | 307.2 | * 85.1 | 100 | 30 | 60 |
| Cocaine-d3 | 4.62 | 307.2 | 185.1 | 27 | 20 | 60 |
| Codeine | 3.24 | 300.2 | * 152.1 | 100 | 55 | 79 |
| Codeine | 3.24 | 300.2 | 165.1 | 102 | 42 | 79 |
| Codeine-d3 | 3.24 | 303.2 | * 165.1 | 100 | 41 | 73 |
| Codeine-d3 | 3.24 | 303.2 | 215.1 | 94 | 26 | 73 |
| Diazepam | 7.02 | 285.1 | * 154.0 | 100 | 27 | 47 |
| Diazepam | 7.02 | 285.1 | 193.0 | 89 | 32 | 47 |
| Diazepam-d5 | 6.99 | 290.1 | * 154.1 | 100 | 28 | 47 |
| Diazepam-d5 | 6.99 | 290.1 | 198.1 | 83 | 33 | 47 |
| EDDP | 5.89 | 278.2 | * 234.2 | 100 | 31 | 71 |
| EDDP | 5.89 | 278.2 | 249.1 | 40 | 24 | 71 |
| EDDP-d3 | 5.89 | 281.2 | * 234.1 | 100 | 32 | 78 |
| EDDP-d3 | 5.89 | 281.2 | 249.1 | 41 | 25 | 78 |
| Ketamine | 4.10 | 238.1 | * 125.0 | 100 | 28 | 43 |
| Ketamine | 4.10 | 238.1 | 220.1 | 49 | 15 | 43 |
| Ketamine-d4 | 4.09 | 242.0 | * 129.0 | 100 | 28 | 50 |
| Ketamine-d4 | 4.09 | 242.0 | 224.1 | 50 | 16 | 50 |
| MDMA | 3.59 | 194.1 | * 105.1 | 100 | 24 | 38 |
| MDMA | 3.59 | 194.1 | 163.1 | 38 | 13 | 38 |
| MDMA-d5 | 3.58 | 199.1 | * 107.0 | 100 | 25 | 38 |
| MDMA-d5 | 3.58 | 199.1 | 165.1 | 34 | 13 | 38 |
| Methadone | 6.47 | 310.2 | * 105.0 | 100 | 27 | 51 |
| Methadone | 6.47 | 310.2 | 265.2 | 38 | 15 | 51 |
| Methadone-d3 | 6.46 | 313.2 | * 105.0 | 100 | 29 | 55 |
| Methadone-d3 | 6.46 | 313.2 | 268.2 | 35 | 16 | 55 |
| Methamphetamine | 3.39 | 150.1 | * 91.1 | 100 | 20 | 35 |
| Methamphetamine | 3.39 | 150.1 | 119.1 | 40 | 11 | 35 |
| Methamphetamine-d5 | 3.37 | 155.1 | * 92.0 | 100 | 20 | 34 |
| Methamphetamine-d5 | 3.37 | 155.1 | 121.0 | 61 | 12 | 34 |
| Zopiclone | 4.18 | 389.1 | * 217.0 | 100 | 33 | 44 |
| Zopiclone | 4.18 | 389.1 | 245.0 | 57 | 17 | 44 |
| Zopiclone-d4 | 4.18 | 393.2 | * 217.0 | 100 | 32 | 40 |
| Zopiclone-d4 | 4.18 | 393.2 | 245.0 | 64 | 20 | 40 |

| **Supplemental Table 6. Summary of analytical validation parameters evaluated for LC-MS/MS.** | | | | | | | | | |
| --- | --- | --- | --- | --- | --- | --- | --- | --- | --- |
| Compound | Bias (%) | | | Precision (% CV) | | | R^2^ | LOD *^a^* (ng/mL) | LOQ *^b^* (ng/mL) |
|  | QC Low | QC Med | QC High | QC Low | QC Med | QC High |  |  |  |
| 6-MAM | -8 | -3 | -7 | 4 | 2 | 2 | 0.9881 | 1.3 | 3.9 |
| Alprazolam | -5 | -5 | -10 | 4 | 4 | 3 | 0.9797 | 9 | 27.4 |
| Buprenorphine | -10 | -2 | -6 | 3 | 3 | 3 | 0.9852 | 1.5 | 4.4 |
| BZE | -7 | -6 | -7 | 7 | 6 | 7 | 0.9748 | 14.8 | 44.9 |
| Cocaine | -8 | -6 | -3 | 4 | 3 | 3 | 0.9828 | 6.8 | 20.6 |
| Codeine | -8 | -5 | -4 | 3 | 4 | 3 | 0.9854 | 5.9 | 17.8 |
| Diazepam | -4 | -5 | -12 | 3 | 3 | 3 | 0.9812 | 11.3 | 34.4 |
| EDDP | -8 | -5 | -2 | 3 | 3 | 3 | 0.9826 | 6.7 | 20.2 |
| Ketamine | -1 | -5 | -13 | 3 | 3 | 4 | 0.9812 | 12.1 | 36.7 |
| MDMA | -8 | -4 | -2 | 3 | 4 | 3 | 0.9838 | 6.6 | 20.1 |
| Methadone | -7 | -3 | -10 | 4 | 3 | 4 | 0.9838 | 10.6 | 32 |
| Methamphetamine | -5 | -4 | -3 | 4 | 3 | 3 | 0.9828 | 6.9 | 21 |
| Zopiclone | -8 | -5 | -8 | 3 | 4 | 3 | 0.9835 | 10.3 | 31.1 |
| *^a^* Limit of detection (LOD) was calculated from the standard deviation of the y-intercept (s_y_) and the average slope (Avg_m_) using the following equation: (3.3 × s_y_) / Avg_m_  *^b^* Limit of quantification (LOQ) was calculated from the standard deviation of the y-intercept (s_y_) and the average slope (Avg_m_) using the following equation: (10 × s_y_) / Avg_m_ | | | | | | | | | |

| **Supplemental Table 7. Summary of analytical validation parameters evaluated for PS-MS.** | | | | | | | | | |
| --- | --- | --- | --- | --- | --- | --- | --- | --- | --- |
| Compound | Bias (%) | | | Precision (% CV) | | | R^2^ | LOD *^a^* (ng/mL) | LOQ *^b^* (ng/mL) |
|  | QC Low | QC Med | QC High | QC Low | QC Med | QC High |  |  |  |
| 6-MAM | -15 | 0 | -9 | 4 | 10 | 6 | 0.9850 | 3.9 | 12 |
| Alprazolam | -5 | -3 | 0 | 4 | 9 | 6 | 0.9958 | 13.7 | 41.4 |
| Buprenorphine | -13 | 2 | -4 | 14 | 22 | 21 | 0.9809 | 9.0 | 27.3 |
| BZE | 8 | 6 | 4 | 3 | 9 | 5 | 0.9931 | 43.1 | 131 |
| Cocaine | 8 | 6 | 5 | 3 | 9 | 5 | 0.9937 | 49.0 | 149 |
| Codeine | -9 | -3 | 3 | 4 | 9 | 5 | 0.9959 | 29.8 | 90.2 |
| Diazepam | 10 | 6 | 0 | 3 | 10 | 7 | 0.9916 | 44.4 | 135 |
| EDDP | 7 | 5 | -1 | 3 | 9 | 5 | 0.9941 | 8.2 | 25 |
| Ketamine | 8 | 5 | 4 | 3 | 9 | 6 | 0.9937 | 40.3 | 122 |
| MDMA | 7 | 5 | -1 | 3 | 9 | 6 | 0.9940 | 8.8 | 26.7 |
| Methadone | -5 | -3 | 0 | 3 | 10 | 6 | 0.9970 | 20.2 | 61.3 |
| Methamphetamine | 8 | 6 | -1 | 3 | 9 | 5 | 0.9940 | 8.5 | 25.6 |
| Zopiclone | -10 | -2 | 1 | 4 | 8 | 5 | 0.9967 | 18.5 | 56.2 |
| *^a^* Limit of detection (LOD) was calculated from the standard deviation of the y-intercept (s_y_) and the average slope (Avg_m_) using the following equation: (3.3 × s_y_) / Avg_m_  *^b^* Limit of quantification (LOQ) was calculated from the standard deviation of the y-intercept (s_y_) and the average slope (Avg_m_) using the following equation: (10 × s_y_) / Avg_m_ | | | | | | | | | |

| **Supplemental Table 8. Comparison of ISTD responses between plasma and serum for LC-MS/MS and PS-MS.** | | | | | | | |
| --- | --- | --- | --- | --- | --- | --- | --- |
| Method | Matrix | Mean log_10_ ISTD response | Mean % CV of ISTD response | Normality test  p-value | T-test  p-value *^a^* | F-test  p-value *^b^* | Ionisation effects *^c^* |
| LC-MS/MS | Plasma | 8.00 | 15 | 0.06 | 0.01 | 0.36 | -2 to -63 % |
|  | Serum | 7.85 | 21 | 0.25 |  |  | 3 to 68 % |
| PS-MS | Plasma | 8.05 | 25 | 0.48 | 0.09 | 0.40 | -46 to -80% |
|  | Serum | 7.98 | 26 | 0.30 |  |  | -54 to -86 % |
| *^a^* Two-sample t-tests assuming unequal variances, reported values are two-tailed P(T≤t).  *^b^* Two-sample F-test for variances, reported values are one-tailed P(F≤f).  *^c^* Calculated from the following equation: (average area of ISTD spiked matrix / average area of ISTD spiked neat standards – 1) × 100. Values > 0 % indicate ion enhancement; values < 0 % indicate ion suppression. | | | | | | | |

| **Supplemental Table 9. Screening performance metrics per analyte for PS-MS relative to LC-MS/MS. Metrics that could not be calculated due to absence of detected positive samples for the analyte are indicated by "-".** | | | | | | | | |
| --- | --- | --- | --- | --- | --- | --- | --- | --- |
| Analytes | TP | TN | FP | FN | Sensitivity | Specificity | PPV | NPV |
| 6-MAM | 0 | 64 | 0 | 0 | - | 100% | - | 100% |
| Alprazolam | 3 | 61 | 0 | 0 | 100% | 100% | 100% | 100% |
| Buprenorphine | 0 | 64 | 0 | 0 | - | 100% | - | 100% |
| BZE | 17 | 42 | 0 | 5 | 77.3% | 100% | 100% | 89.4% |
| Cocaine | 0 | 62 | 0 | 2 | 0% | 100% | - | 96.9% |
| Codeine | 2 | 62 | 0 | 0 | 100% | 100% | 100% | 100% |
| Diazepam | 5 | 52 | 0 | 7 | 41.7% | 100% | 100% | 88.1% |
| EDDP | 0 | 64 | 0 | 0 | - | 100% | - | 100% |
| Ketamine | 11 | 53 | 0 | 0 | 100% | 100% | 100% | 100% |
| MDMA | 6 | 58 | 0 | 0 | 100% | 100% | 100% | 100% |
| Methadone | 1 | 63 | 0 | 0 | 100% | 100% | 100% | 100% |
| Methamphetamine | 22 | 42 | 0 | 0 | 100% | 100% | 100% | 100% |
| Zopiclone | 0 | 64 | 0 | 0 | - | 100% | - | 100% |

| **Supplemental Table 10. Distribution of PS-MS concentrations for analytes detected in the screening-only cohort. Analytes with no detectable PS-MS signal are indicated by "-".** | | | |
| --- | --- | --- | --- |
| Analyte | Median (ng/mL) | Minimum concentration (ng/mL) | Maximum concentration (ng/mL) |
| 6-MAM | - | - | - |
| Alprazolam | - | - | - |
| Buprenorphine | - | - | - |
| BZE | 726 | 275 | 2,250 |
| Cocaine | - | - | - |
| Codeine *^a^* | 161 | 161 | 161 |
| Diazepam | 313 | 182 | 2,320 |
| EDDP | 65.3 | 62.8 | 67.7 |
| Ketamine | 462 | 162 | 1,650 |
| MDMA | 172 | 30.0 | 1,170 |
| Methadone | 370 | 61.4 | 634 |
| Methamphetamine | 150 | 49.7 | 1,370 |
| Zopiclone | - | - | - |
| *^a^* Only a single detection was observed for this analyte; median, minimum and maximum values are therefore identical. | | | |
|  |  |  |  |
